# Effect of Surface Active Agent (SAA) on 50/70 Bitumen Foaming Characteristics

**DOI:** 10.3390/ma12213514

**Published:** 2019-10-26

**Authors:** Mateusz M. Iwański, Anna Chomicz-Kowalska, Krzysztof Maciejewski

**Affiliations:** 1 Department of Building Engineering Technologies and Organization, Faculty of Civil Engineering and Architecture, Kielce University of Technology, Al. Tysiąclecia Państwa Polskiego 7, 25-314 Kielce, Poland; matiwanski@tu.kielce.pl; 2 Department of Transportation Engineering, Faculty of Civil Engineering and Architecture, Kielce University of Technology, Al. Tysiąclecia Państwa Polskiego 7, 25-314 Kielce, Poland; kmaciejewski@tu.kielce.pl

**Keywords:** foamed bitumen, surface active agent, expansion ratio, half-life, half-warm mix asphalt

## Abstract

To ensure the standard properties of half-warm asphalt (HWA) mixes produced with foamed bitumen, the binder needs to have the best possible characteristics. One way to attain this is to modify the bitumen before it is foamed. The 50/70 penetration bitumen used in this study, was modified with a surface active agent (SAA) at different rates (0.2%, 0.4%, and 0.6% by wt.). The effect of the modifier on the bitumen properties (penetration, softening point, the Fraass breaking point, dynamic viscosity at 60 °C, 90 °C, and 135 °C) and on the binder foaming parameters (expansion ratio - ER, half-life - HL, foam index - FI) was investigated and the optimum quantity of foaming water was determined. Statistical analysis of the results showed that the addition of 0.6% SAA had the most beneficial effect on the set of 50/70 bitumen standard properties and foaming characteristics.

## 1. Introduction

Currently, the majority of asphalt road surfaces are made of traditional hot bituminous mixtures (HMA). A classical layer of HMA is produced at high processing temperatures that can reach up to 180 °C, depending on the type of bitumen used [1,2]. 

Increased awareness of the need to improve sustainability and lessen the impact on the environment has been observed in the road construction industry since the beginning of the 21st century [3]. New technologies have been developed that contribute to the reduction in energy intensity of construction processes and the emission of greenhouse gases. One of the solutions found, Warm Mix Asphalt (WMA) technology [4,5,6], allows lowering the mixing and paving temperatures. The technology has evolved in two main directions. One of these directions is focused on the use of additives acting on the viscosity of the bitumen such that the mix production temperature can be reduced [7,8,9]. The most representative additive in this group is the Fischer-Tropsch (F-T) synthetic wax [10,11,12]. Not only does it reduce the mixing and paving temperature by 20 °C to 30 °C, but it also improves the standard [13] and rheological properties of the binder [14,15] and enhances the mix mechanical characteristics, such as stiffness modulus or resistance to permanent deformations [16]. Adding F-T wax has been proved to extend the service life of road surfaces [17,18].

The second direction makes use of foamed bitumen in the process of manufacturing bituminous mixes. The binder in the form of a foam coat the mineral mix particles at a much lower temperature than conventional binders [19]. Aggregates are to be dried at a temperature not higher than 100 °C to allow the particle pores to retain some membrane water thereby providing additional bitumen foaming [20]. Bitumen is foamed in two ways: either by adding zeolite to the mix [21] or by water acting on the binder [22]. Due to its chemical structure, zeolites are included in the group of aluminosilicates. They can be organic [23] or synthetic that is derived from the processing of various by-products, such as dust, generated in technological processes [24]. The crystalline structure of zeolites is responsible for their high absorption of water [24], which while being released gradually foams the binder in contact with hot bitumen [25]. The WMA technology produces bituminous mixtures at temperatures of about 120 °C. A technology that allows lowering the mix production temperature even further to around 100 °C is labelled half warm mix asphalt (HWA) and makes use of bitumen foamed with water [26,27,28]. The foaming process involves injecting cold water into hot bitumen at high pressure in an expansion chamber [19,29]. As a result, the water evaporates rapidly and produces the foam of reduced viscosity having the ability to coat the aggregate at a much lower temperature than in the traditional method [30]. 

The process of water-based foaming was originally applied in cold recycling to produce base course mixes [30,31]. Successful performance of foamed bitumen in the lower layers of the pavement structure encouraged researchers to search for solutions that would allow the technology to be implemented in upper layer mixes [32]. To ensure that the properties of foamed bitumen mixes are comparable to those of classical HMA, the quality of the binder, described with the foaming parameters such as half-life (HL) and expansion ratio (ER), must be high [19,29]. The requirements for foam characteristics (ER - 8 and HL - 10 s) developed at the turn of the 20th and 21st centuries were established for cold recycling binders [19,29]. The foam characteristics of the HWA binder should have higher values to meet the higher requirements set forth for the bituminous mixes intended for the upper layers of the pavement structure. For this purpose, before foaming the bitumen was modified by adding various types of additives. Favourable results were obtained with the addition of Fischer-Tropsch (F-T) synthetic wax. The F-T wax was found to significantly enhance the bitumen foam characteristics [33,34], reduce the viscosity of the bitumen within the operating temperature range [35], improve the rheological parameters of the binder [36] and increase the resistance of the bituminous mix to permanent deformations [37,38].

A disadvantage observed in the effects of F-T wax application is the limited reduction in compaction temperatures. Below 90 °C, F-T wax is released from the binder in the form of crystallites [39]. This makes compaction very difficult or impossible and the adequate quality of the pavement layer may not be achieved. In countries with a moderate climate, low ambient temperatures in the early spring and late autumn may prevent or restrict the use of F-T wax-based technology. Other types of asphalt additives are being tested for improved foam parameters, mix quality, and extended paving season [40]. 

One of the often-overlooked aspects in the investigations regarding foamed bitumen and foamed bitumen mixtures is the effect of widely used adhesion promoters, usually in the form of surface active agents (SAA), on the foamability and properties of different bituminous binders. These agents are often used as a mandatory means for providing adequate moisture resistance and durability of asphalt mixtures, but their effects are rarely investigated. 

The aim of this study was to assess the impact of a surface active agent on the conventional and foaming properties of a 50/70 penetration bitumen and its prospects as a way for improving the foaming performance of the investigated bitumen.

## 2. Materials and Research Methodology

### 2.1. Tested Materials

The bitumen used was a 50/70 penetration grade, commonly used in the countries of central and eastern Europe in bituminous mixes for road pavements under traffic characterized by 2.5 × 10^6^ < ESAL_100 kN_ < 7.3 × 10^6^ (ESAL – equivalent single axle load) [41]. Table 1 summarizes the properties of the binder. 

The SAA added to the 50/70 bitumen before foaming was a mixture of substances contributing markedly to the improved binder-aggregate adhesion. The SAA properties are compiled in Table 2.

### 2.2. Experimental Program

Adequate preparation of the binder samples is very important as it affects the test quality and uniformity of test results. The binder samples were delivered directly from the refinery in 5-litre containers. The samples were then poured into 1-litre steel cans in compliance with EN 12594. The binder was prepared by combining two materials, 50/70 bitumen and SAA. An appropriate amount of additive was added to a 1000 g binder sample. Mixing the binder with the additive involved heating the binder to a temperature 100 °C higher than the softening point and mixing it at a speed of 150 rpm for 30 s and then at 600 rpm for 270 s. The hot binder samples were reduced to 250 g (test sample weight) in compliance with EN 12594. The samples were placed in a heated vacuum chamber to remove air bubbles. Since SAA is a liquid with a density lower than that of bitumen, it was necessary to check macroscopically whether a film of the additive formed on the surface of the binder, in which case the sample was rejected.

The SAA was added at a rate of 0.2%, 0.4% and 0.6% of virgin binder mass. The effect of SAA on the properties of the 50/70 bitumen was investigated in two phases. In the first phase of the present study, the basic and rheological properties of the bitumen containing SAA were examined prior to foaming with 9 replicates per test: penetration at 25 °C (*Pen,* EN 1426:2015-08), softening point (*T_R&B_*, EN 1427:2015-08), Fraass breaking point (*T_Fraass_*, EN 12593:2015-08), dynamic viscosity at 60 °C, 90 °C and 135 °C (*η_60_, η_90_*, *η_135_,* EN 13302:2011).

The penetration index *PI* values were found as in Equation (1) by determining the bitumen temperature susceptibility taking into account its 25 °C penetration grade (*Pen*) and softening point (T_R&B_) and utilizing the formula according to EN 12591:

(1) $$PI=\frac{20\times T_{R\&B}+500\times \log\left(Pen\right)-1952}{T_{R\&B}+50\times \log\left(Pen\right)-120}$$

The calculation was based on the assumption that the penetration of the binder is 800 (0.1 mm) at the softening point *T_PiK,_* and the lower the PI value, the more easily the binder changes its consistency with temperature. 

The temperature range of plasticity (plasticity range PR) of the binder, dependent on its softening point (*T_R&B_*) and breaking point (*T_Fraass_*), was determined by Equation (2) in compliance with the requirements laid down in [2] according to the following formula:

(2) $$PR=T_{R\&B}-T_{Fraass}\;\left({}^{\circ}C\right)$$

The dynamic viscosity of the binder was determined on a Rheotest RN 4 rheometer. Both the binder sample preparation and the tests were carried out as per EN 12702-2.

The 50/70 bitumen foam characteristics of expansion ratio ER [18,28] and half-life HL [19,29] were determined in the second stage of the study with 9 replicates.

Physical properties of the foam were tested in a foaming plant type Wirtgen WLB-10S by applying different foaming water contents (FWC): 1.5%, 2.0%, 2.5%, 3.0%, 3.5% and 4.0% by wt. The conditions of the foam testing were as given in Table 3:

Then, the optimum values of FWC-dependent ER and HL parameters were established from a mathematical relationship. The optimum FWC was also determined. 

The quality of the foamed bitumen was evaluated using Equation (3) in accordance with the recommendation proposed by Jenkins [18] on the basis of the *FI* foaming index, which is measured as the time in seconds and calculated using the following formula:

(3) $$FI=\frac{-\mathrm{HL}}{ln2}\times \left(4-ER_{m}-4ln\times \left(\frac{4}{ER_{m}}\right)\right)+\left(\frac{1+C}{2C}\right)\times ER_{m}\times t_{s}\;\;\left(s\right)$$

where: C–the correction factor (ER_m_/ER_a_), HL–half-life (s), t_s_–bitumen spraying time (s), ER_m_–the measured expansion ratio (immediately after bitumen foaming), ER_a_–the actual expansion ratio.

The results were analysed using Statistica software [42] in order to ensure their reliability and to determine significant dependencies between the tested parameters of the binder and the quantity of SAA used. In the statistical results, red font denotes statistical significance (α = 0.05). 

## 3. Results and Discussion

### 3.1. The Effects of The SAA Content on basic Bitumen Properties 

**Figure 1:**
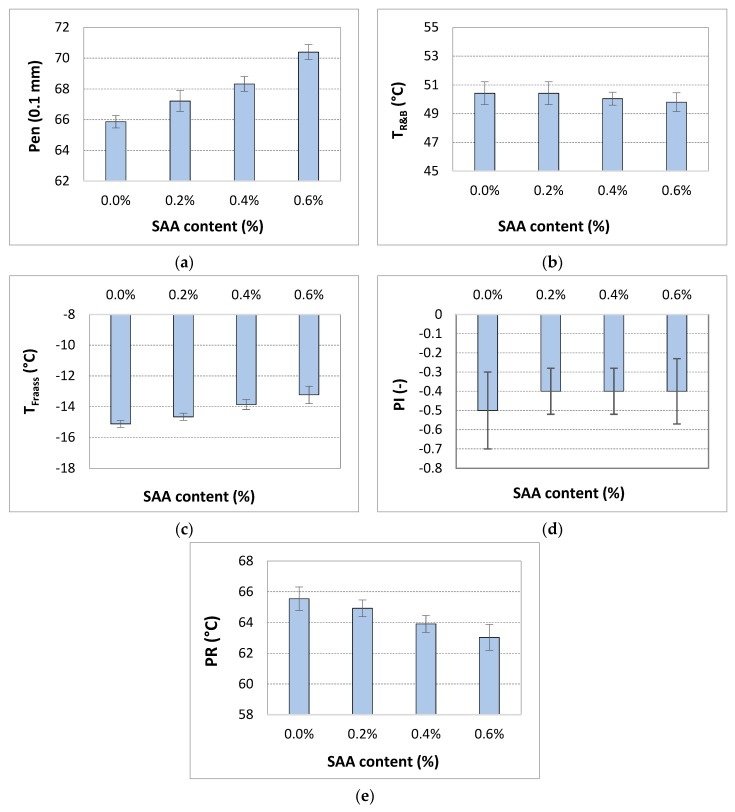
Effect of SAA content on the properties of 50/70 bitumen, **a**) penetration, **b**) softening point, **c**) Fraass breaking point, **d**) penetration index, **e**) plasticity range (error bars represent standard deviation).

The results of the preliminary statistical analysis are compiled in Table 4 and their graphical interpretation is shown in Figure 1.

The influence of SAA on the binder properties was evaluated with a one-way ANOVA variance analysis. The results are presented in Table 5. 

Statistical analysis of the results shown in Figure 1a–e and summarized in Table 4 clearly indicates that SAA content has a significant effect on penetration at 20 °C and Fraass breaking point because the p-value is less than the defined significance level α = 0.05. Only in case of softening point, there is no significant influence of SAA addition to 50/70 bitumen.

The use of the surface active agent has resulted in a slight increase in the penetration of the evaluated bitumen, which gradually changed from 65.9 [0.1 mm] to 70.4 [0.1 mm] with the increasing SAA content. The results show that the softening point was unchanged by the addition of SAA, regardless its concentration. The low temperature property of the bitumen measured by the Fraass breaking point was slightly affected by the addition of SAA in 0.2% concentration, resulting in its 0.4 °C increase. Further addition of the SAA up to 0.6% content resulted in significant changes of Fraass breaking point setting it at −13.2 °C marking a 1.9 °C total difference. Because the changes in penetration and softening point due to addition of SAA were minor, also the penetration index was nearly unaffected by the effects of the additive. A different situation was seen in regard to the plasticity range defined as the difference between the softening point and Fraass breaking point. The addition of SAA caused a small decrease in the PR values, associated mainly with the changes in Fraass breaking point. 

### 3.2. Determining Dynamic Viscosity of The Binder with SAA

Dynamic viscosity is considered an important rheological parameter that has a significant influence on physical and mechanical properties and performance of pavement bituminous layers. The parameter was used to establish the influence of SAA on the changes occurring in the bitumen relative to the reference binder. For this purpose, samples of 50/70 bitumen were prepared with SAA contents ranging from 0.0% to 0.6% of binder mass. The test was performed using the methodology set forth in EN 13302 in a Rheotest rotary viscometer at a shear rate of 1 s^−1^. 

**Figure 2:**
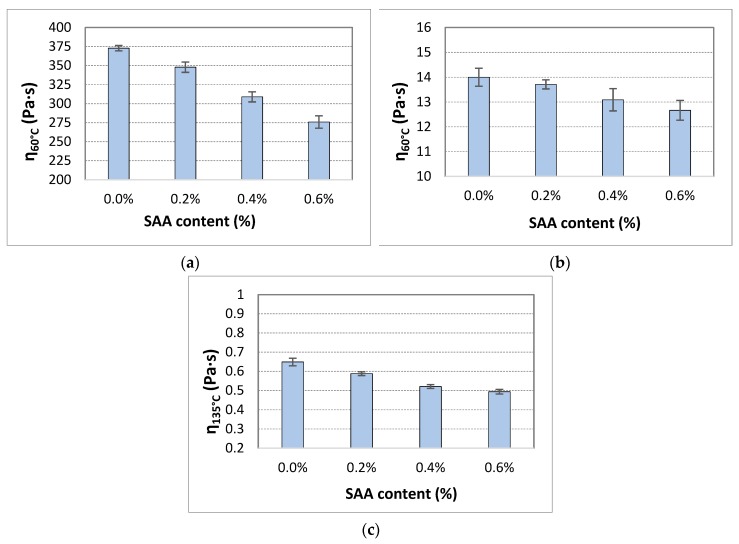
Effect of SAA addition on dynamic viscosity of 50/70 bitumen (box plot) at temperatures **a**) 60 °C, **b**) 90 °C, **c**) 135 °C (error bars represent standard deviation).

The range of dynamic viscosity of 50/70 bitumen with SAA was determined for temperatures 60 °C, 90 °C and 135 °C, which correspond to the significant operating ranges of compaction and binder performance in the pavement layers. Nine samples were used in the tests. The average values of dynamic viscosity at given test temperature and the respective coefficients of variation are tabulated in Table 6. The graphical interpretation is shown in Figure 2.

Analysis of the results of the SAA effect on the dynamic viscosity of 50/70 bitumen shows that dynamic viscosity decreases with increasing SAA content 0.0% to 0.6% across the whole temperature range under analysis. The decrease rate depends on the SAA content and test temperature. At 60 °C the use of 0.6% SAA by binder mass reduced dynamic viscosity of the reference bitumen by approx. 26%. Similarly, at 90 °C, the dynamic viscosity decreased by approx. 10%, and at 135 °C by approx. 24%. The correlation between the SAA content and dynamic viscosity was high in the measured data. The coefficient of determination (equal to the squared value of Pearson correlation coefficient) amounted to over 0.9 in the case of η_60_ and η_135_, and was equal to 0.688 in the case η_90_, thereby indicating a strong correlation.

In order to evaluate the influence of SAA on dynamic viscosity in the temperature range used in this study, a one-way ANOVA variance analysis was performed for each temperature. The ANOVA analysis results for dynamic viscosity at 60 °C, 90 °C and 135 °C are shown in Table 7.

Analysis of the obtained results indicates clearly that the SAA content in 50/70 bitumen is a significant factor influencing its dynamic viscosity at 60 °C, 90 °C, and 135 °C as shown by the p-value is less than the defined significance level α = 0.05. 

**Figure 3:**
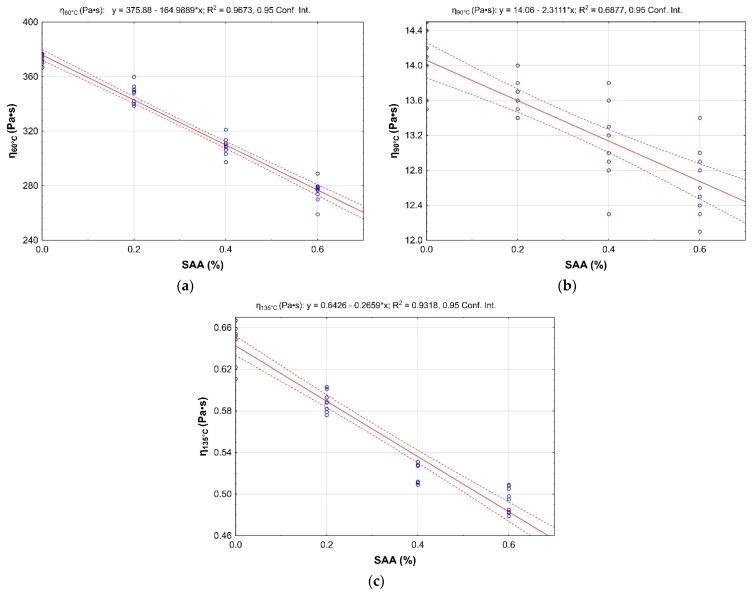
Effect of SAA on 50/70 bitumen viscosity versus test temperature **a**) 60 °C, **b**) 90 °C, and **c**) 135 °C. The plots represent discrete results, linear approximation of the results and the 95% confidence intervals.

The relationship between dynamic viscosity at 60 °C, 90 °C, and 135 °C and the SAA content is shown in Figure 3.

The application of SAA reduces dynamic viscosity of 50/70 bitumen in the studied temperature range. The variation rate is dependent both on the bitumen-additive content proportion and on the test temperature. 

In order to comprehensively describe the change in dynamic viscosity of the 50/70 bitumen in the studied temperature range, the model of the second-degree polynomial (4) was adopted:

η = b_0_ + b_1_ × x_1_ + b_2_ × x_2_ + b_3_ × x_1_ × x_2_ + b_4_ × x_1_^2^ + b_2_ × x_2_^2^ (4)

where: x_1_ = SAA (%), x_2_ = Temp (°C), b_0_ − b_5_ – regression coefficients.

In the first phase of the evaluation of the model, a statistical significance test was performed in an analysis of variance (ANOVA) via Statistica (Table 8).

Analysis of the parameters listed in Table 7 shows clearly that the SAA content and test temperature are important factors having effects on the dynamic viscosity of 50/70 bitumen, as the p-value is less than the assumed significance level α = 0.05. It can also be concluded that there is an interaction between the SAA content and the test temperature which affects the dynamic viscosity of the binder (the p-value is less than α = 0.05). A regression model of the dependence of dynamic viscosity of 50/70 bitumen on the SAA content and test temperature was developed. The values describing the model are summarized in Table 9.

The procedure performed disclosed that the value of the corrected coefficient of determination R^2^ is more than 99%, which indicates that the model is adequate. Dynamic viscosity of 50/70 bitumen is significantly influenced by the SAA quantity used, test temperature and the interaction of these two factors.

**Figure 4:**
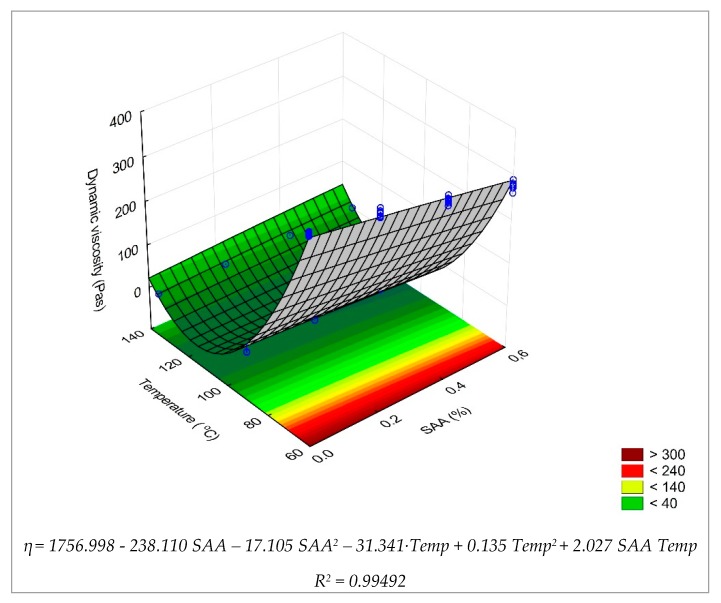
Dynamic viscosity of 50/70 bitumen versus test temperature and SAA content. The provided equation describes the relationship between the SAA content, temperature, and bitumen dynamic viscosity.

The graphical interpretation of dynamic viscosity variability in terms of SAA content and test temperature is shown in Figure 4 via the response surface. 

Analysis of the results in Figure 4 confirms that with the increase in the test temperature, the dynamic viscosity of the 50/70 bitumen decreases over the whole experiment range. This is in line with the general trend for bitumen binders. In the temperature range above 100 °C, the low viscosity of the 50/70 bitumen will ensure adequate coating of the mineral mix particles during mix production. Within a range of summer temperatures, because of its high viscosity, the bitumen will contribute to ensuring adequate resistance of the pavement to permanent deformation. The SAA affects the viscosity of 50/70 bitumen. The increased content of SAA reduces the value of this parameter over the whole range of test temperatures. This may be considered as an adverse effect but because the viscosity reduction is only slight, the characteristics of the bituminous mixture made with this binder should remain nearly unaffected. 

### 3.3. Foamed Bitumen Properties

**Figure 5:**
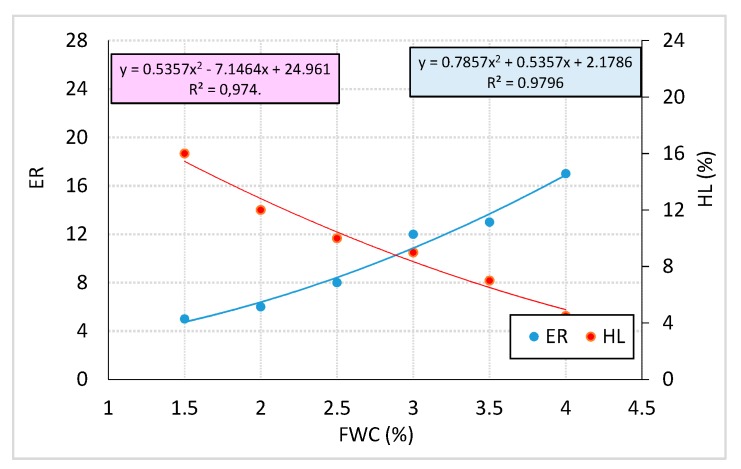
Foamed 50/70 bitumen characteristics.

The objective of the second phase of the present study was to determine the effect of SAA on the value of maximum ER and half-life HL of 50/70 bitumen. The SAA was applied at 0.2%, 0.4%, and 0.6% of the binder mass. The maximum amount of SAA dosed was 0.6% by bitumen mass, as this value was recommended by the manufacturer as the limit value between applications in “hot” and “cold” bitumen mixes. The foaming characteristics (ER, HL) of the reference bitumen expressed as the average values from 9 determinations are shown in Figure 5.

**Figure 6:**
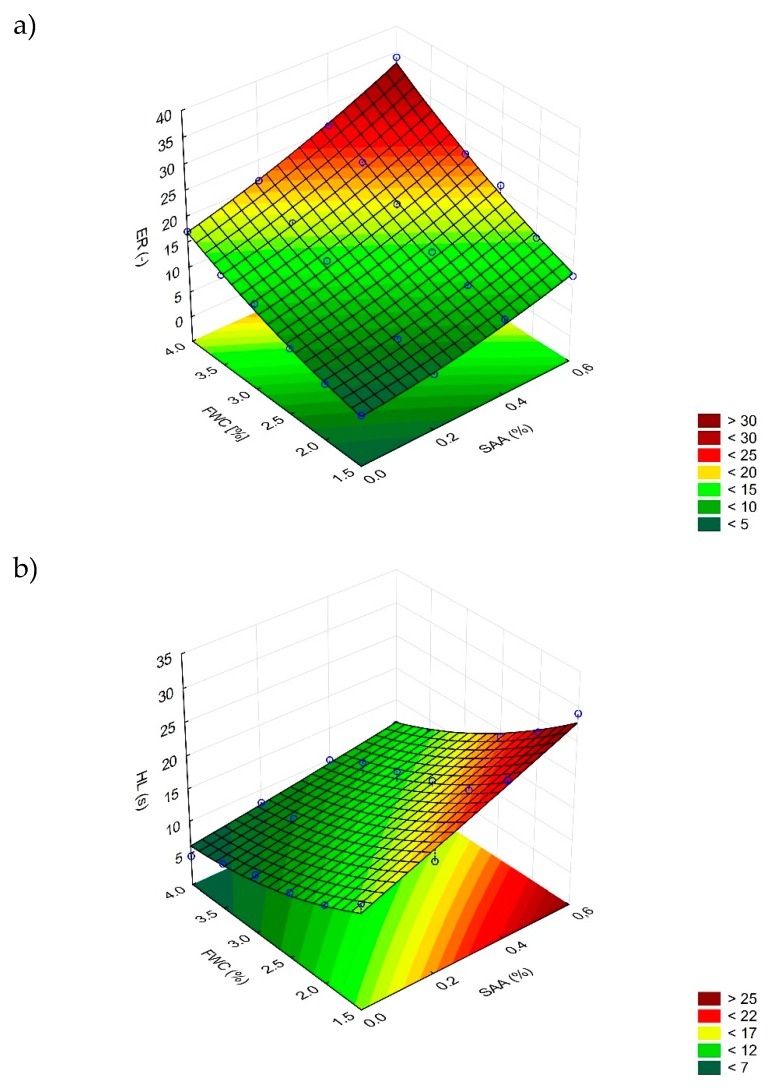

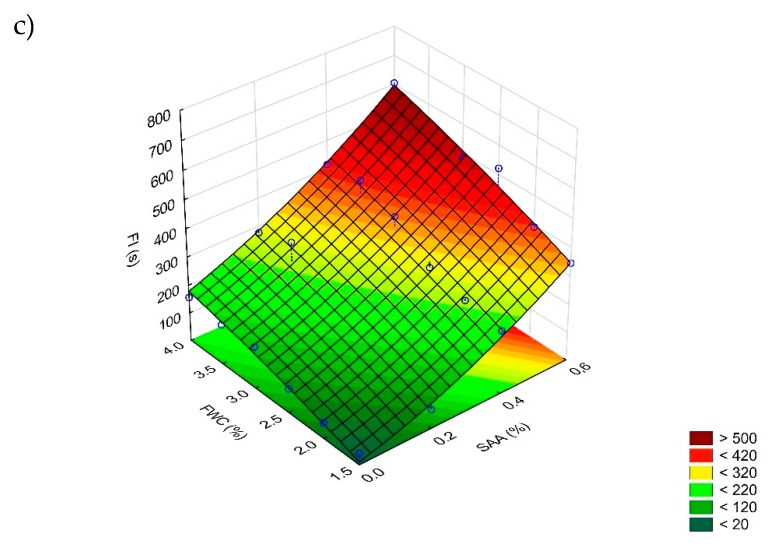
Response surface of the maximum (**a**) expansion ratio (ER), (**b**) half-life (HL) and (**c**) foam index FI against SAA content and foaming water content foaming water contents (FWC).

Figure 6 shows the obtained foamed 50/70 binder characteristics (ER, HL, FI) versus FWC and SAA content. Statistical analysis of these variables is presented in Table 10, while the estimation of the model parameters is given in Table 11.

Statistical analysis showed that all the evaluated responses (ER, HL, FI) were influenced by both independent variables: the foaming water content and the quantity of the surface active agent. In all cases both linear main effects were statistically significant. What is more, in the case of expansion ratio and half-life, the interactions also obtained p-values smaller than the assumed significance level (α = 0.05). In the evaluation of half-life and foam index, additionally quadratic effects of FWC and SAA content, respectively, were statistically significant. All effects in the assessment of expansion ratio resulted in p-values < 0.05.

Analysis of the obtained foamed 50/70 bitumen characteristics in terms of the quantity of SAA used shows its positive influence. The maximum expansion of the binder and the half-life of the bitumen foam increased with the increasing content of SAA. This binder will be efficient in coating the aggregate particles during mixing, thereby ensuring the mix durability and proper performance. 

**Figure 7:**
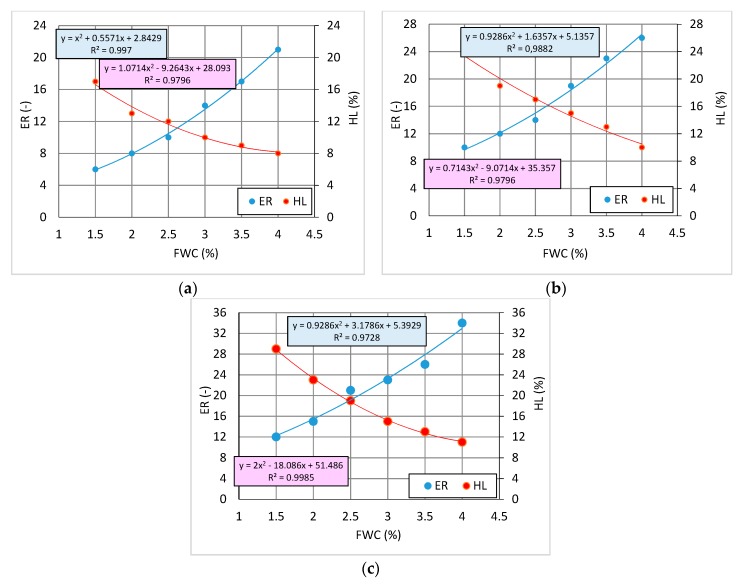
Foaming characteristics of bitumen 50/70 + 0.2% SAA (**a**), 50/70 + 0.4% SAA (**b**), 50/70 + 0.6% SAA (**c**).

Figure 7 shows the plots of foamed 50/70 bitumen characteristics versus foaming water contents for the three evaluated SAA contents. The optimum FWC’s were established at the intersection of expansion ratio and bitumen foam half-live curves. 

The results confirmed the positive influence of SAA on the foamed 50/70 bitumen characteristics. By reducing the bitumen surface tension, the SAA addition enhanced its foaming capability. 

**Figure 8:**
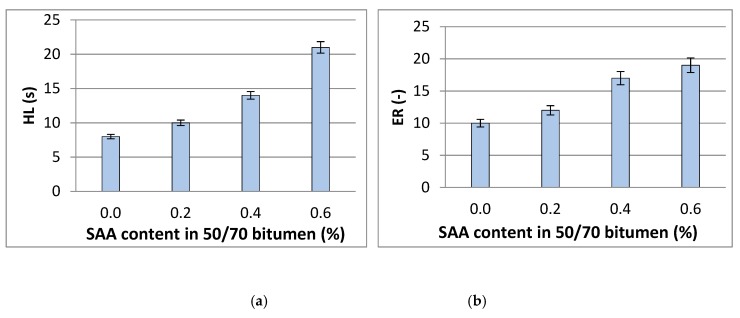
Average values of foaming parameters of 50/70 bitumen with additives, **a**) half-life–HL, **b**) maximum expansion–ER (error bars represent standard deviation).

Average values of the obtained foaming characteristics with respect to the type of bitumen and quantity of the additive used are summarised in Table 12 and shown graphically in Figure 8. 

Analysis of the results indicates that the S AA contributes to the increase in the values of foamed 50/70 bitumen characteristics. The rate of ER and HL variation is dependent on the quantity of dosed additive and increases with the SAA concentration in the binder. The most favourable foam characteristics were obtained when 0.6% SAA by bitumen mass was applied. The value of ER almost doubled and the value of HL increased by a factor of 2.5 after adding 0.6% SAA.

Bitumen 50/70 with 0.6% of SAA has the most favourable foam parameters: ER–19, HL–21s and FWC–2.5%. These characteristics are better than those obtained from the recommended modification of 50/70 bitumen with F-T synthetic wax at 2.5% with respect to the binder (ER–18, HL–15s and FWC–2.0%) [35].

### 3.4. Optimisation of The SAA Content with Respect to The Standard Bitumen Properties 

In order to determine the recommended quantity of SAA for the most favourable standard properties of 50/70 bitumen, Statistica software was used [42]. 

The following important parameters of 50/70 bitumen with the addition of SAA at 0%, 0.2%, 0.4% and 0.6% were evaluated:

- softening point (*T_PiK_*),
- Fraass breaking point (*T_Fraass_*),
- penetration index (PI),
- dynamic viscosity at 90 °C (*η_90_*) and 135 °C (*η_135_*),
- maximum expansion (ER),
- half-life (HL).

The characteristics of the models describing the interactions among the binder parameters with respect to the SAA content are tabulated in Table 13. 

The following equations (5-11) were determined in Statistica [42] to describe the changes in 50/70 bitumen properties with respect to SAA content:

T_R&B_ = 50.4477 − 0.2055 × SAA (%) – 1.5277 × SAA (%)^2^ (5)

T_Fraass_ = -15.147778 + 2.5666 × SAA (%) + 1.1111 × SAA (%)^2^ (6)

η_90_ = 14.0266 – 1.8111 × SAA (%) – 0.8333 × SAA (%)^2^ (7)

η_135_ = 0.6510 – 0.3930 × SAA (%) + 0.2118 × SAA (%)^2^ (8)

PI = 0.6744 – 6.1833 × SAA (%) + 7.3611 × SAA (%)^2^ (9)

ER = 9.1777 + 18.6666 × SAA (%) – 2.7777 × SAA (%)^2^ (10)

HL = 8.1611 + 3.5833 × SAA (%) + 29.8611 × SAA (%)^2^ (11)

For assessing the performance characteristics of the binders, the best values of the characteristic were assigned a number of 1 and the least acceptable value was set at 0. Intermediate values ranged from 0 to 1 on a linear scale in line with the methodology described in [43]. The following criteria for the independent binder parameters were applied:

- softening point *T_R&B_* (max–1, min–0),
- Fraass breaking point T_Fraass_ (max–0, min–1),
- penetration index *PI* (max–1, min–0),
- dynamic viscosity at *η_90_* and *η_135_* (max–0, min–1),
- expansion ratio *ER* (max–1, min–0),
- half-life HL (max–1, min–0).

**Figure 9:**
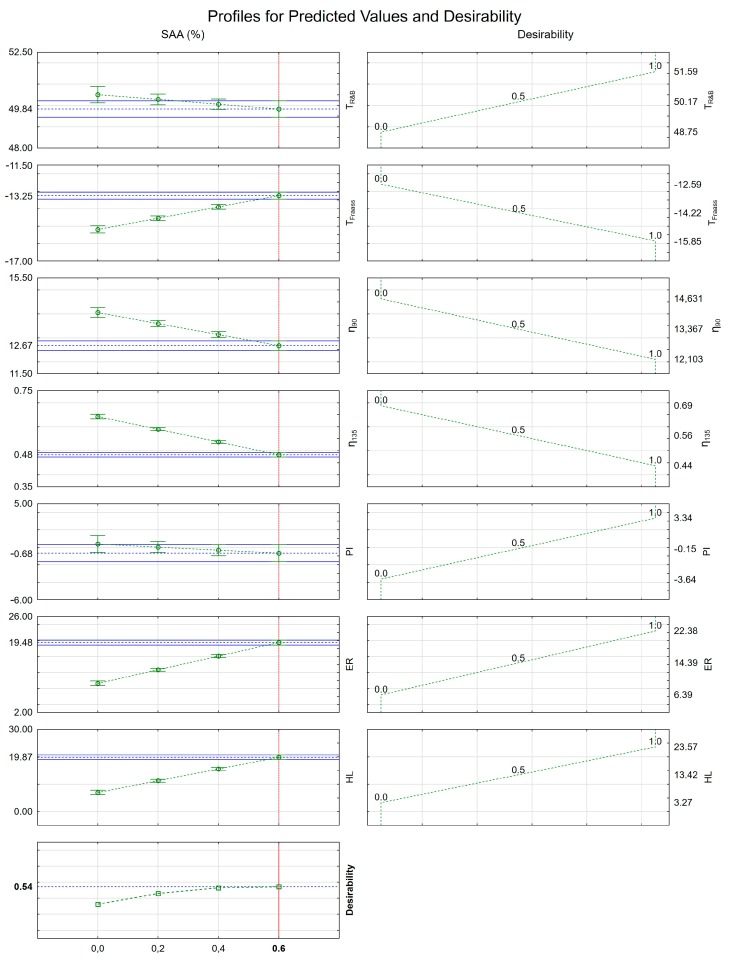
Determination of the best performing content of SAA for the best possible properties of 50/70 binder.

Then the values of usability function were calculated. The results in the form of approximated values and usability profiles are presented graphically in Figure 9.

The results of the optimization show that the addition of the SAA had different impacts on the evaluated binder properties in terms of their desirability. The softening point and Fraass breaking point were negatively affected by the additive, but these changes should not be considered overly significant. All remaining properties elicited on the increased SAA content. The dynamic viscosities measured at 90 °C and 135 °C decreased after incorporating the additive in larger amounts, contributing to possible improvements mix coating and workability. The penetration index after the initial 0.2% addition of the SAA was hardly changed by its further increased concentration. As it was shown in the assessment of the foaming performance, the SAA has significantly supplemented the expansion ratio and bitumen foam half-life exhibited by the 50/70 bitumen, which should translate into improved workability and mixture homogeneity. 

The combined assessment of the SAA effects on the overall performance of the 50/70 bitumen intended for use in half-warm asphalt mixes with foamed bitumen is given by the calculated desirability profile. As shown in Figure 9, the desirability values increased with the increase of the SAA content, reaching a maximum value of 0.543 at 0.6% concentration of the surface active agent. 

Therefore, the analysis of desirability profiles shows that the best performing SAA content in 50/70 bitumen is 0.6% by bitumen mass, in which case the binder parameters reach the highest levels in overall. 

## 4. Conclusions

In this paper, the effects of surface active agent (SAA) on the properties of 50/70 penetration bitumen used in foamed bitumen process were investigated. The analysis of the results showed that the SAA content affected the tested 50/70 bitumen characteristics in different ways depending on the evaluated properties:

- the penetration of the bitumen has increased consistently with the increase of the SAA from 65.9 [0.1 mm] of the base bitumen by as much as 4.5 [0.1 mm] in the bitumen with 0.6% SAA content.
- the Fraass breaking has also gradually increased in the function of the SAA content resulting in 1.9 °C change at 0.6% SAA content,
- there were no significant changes in softening point and penetration index associated with the addition of surface active agent,
- the SAA has reduced the bitumen dynamic viscosity in the measured range of temperatures (60 °C, 90 °C, and 135 °C),
- the addition of SAA resulted in profound improvement of foaming characteristics of 50/70 bitumen, showing increased values of half-life (HL), expansion ratio (ER) and foam index (FI) resulting in possible significant contribution of mixture coating and workability,
- the analysis of the desirability function has shown that the addition of surface active agent in 0.6% concentration should result in superior overall performance of the 50/70 penetration binder enhancing the HWA mix quality and workability, without significantly affecting the binder rheology.

In conclusion, the SAA will have a positive effect on the 50/70 bitumen foaming process and thus on its use in HWA. Its influence on the material characteristics of the bituminous mix, in particular on the resistance to permanent deformations, may vary, in which case it will be necessary to use another additive, such as hydrated lime, which favourably influences the mechanical characteristics of HWA mixtures. The future work in this field will include the verification of the aforementioned results based on investigations of half-warm asphalt mixtures with foamed bitumen in terms of their compactability, mechanical performance, and moisture resistance. 

## Figures and Tables

**Table 1:** Basic properties of 50/70 bitumen.

Property	Test Method	Unit of Measure	Result
Penetration at 25 °C	EN 1426	0.1 mm	65.9
Softening point T_R&B_	EN 1427	°C	50.4
Fraass breaking point	EN 12593	°C	−15.1
Dynamic viscosity at: 60 °C 90 °C 135 °C	EN 12702-2	Pa∙s	372.9 14.0 0.649

**Table 2:** Characteristics of the surface active agent surface active agent (SAA).

Property	Unit of Measure	Value
Appearance	-	Brown viscous liquid
Density at 20 °C	Mg/m^3^	0.98
Pour point	°C	<0
Viscosity at 20 °C	mP	3000
Viscosity at 50 °C	mP	400
Amine index	mg HCl/g	159–185
Acid index	mg KOH/g	<10
Freezing point	°C	<0
Flash point (open flame)	°C	>218

**Table 3:** Bitumen foaming conditions.

Foaming Process Parameters	Value
Bitumen temperature	155 °C
Water temperature	20 °C
Water flow rate	100 g/s
Bitumen foaming time	5 s
Air pressure	500 kPa
Water pressure	600 kPa

**Table 4:** Statistical values of 50/70 bitumen properties versus SAA content.

SAA Content in 50/70 Bitumen (%)	Variable	Value of Statistical Parameters of 50/70 Bitumen Properties					
		Valid N	Max.	Min.	Mean	Std. Dev.	Coef. Var. (%)
0.0	*Pen*	9	66.1	65.4	65.9	0.40	0.6
	*T_R&B_*	9	51.7	49.8	50.3	0.44	0.9
	*T_Fraass_*	9	−15.4	−14.7	−15.1	0.22	1.5
	*PI*	9	−0.6	−0.4	−0.5	0.20	4.6
	*PR*	9	66.9	64.6	65.5	0.77	1.2
0.2	*Pen*	9	68.8	66.1	67.2	0.68	1.0
	*T_R&B_*	9	50.8	49.7	50.3	0.44	0.9
	*T_Fraass_*	9	−15.0	−14.4	−14.7	0.23	1.6
	*PI*	9	−0.6	−0.3	−0.4	0.12	2.0
	*PR*	9	65.7	64.2	64.9	0.53	0.8
0.4	*Pen*	9	68.9	67.7	68.3	0.48	0.7
	*T_R&B_*	9	50.7	49.2	50.0	0.45	0.9
	*T_Fraass_*	9	−14.3	−13.7	−13.9	0.32	2.3
	*PI*	9	−0.5	−0.4	−0.4	0.12	2.8
	*PR*	9	64.6	63.2	63.9	0.54	0.8
0.6	*Pen*	9	70.9	69.6	70.4	0.54	0.8
	*T_R&B_*	9	50.9	49.0	49.8	0.65	1.3
	*T_Fraass_*	9	−13.7	−12.3	−13.2	0.57	4.3
	*PI*	9	−0.5	−0.4	−0.4	0.17	4.2
	*PR*	9	64.6	62.0	63.0	0.84	1.3

**Table 5:** Evaluation of significant influence (ANOVA) of the SAA factor on *Pen*, *T_R&B_*, and *T_Fraass_*.

Response	SS	MS	F	p-Value
*Pen*	98.8	32.9	119.8	<0.001
*T_R&B_*	2.52	0.84	1.8	0.171
*T_Fraass_*	19.007	6.336	48.09	<0.001

**Table 6:** Mean values of 50/70 bitumen dynamic viscosity versus SAA content and test temperature (60 °C, 90 °C, and 135 °C).

SAA Content in 50/70 Bitumen (%)	Dynamic Viscosity (Pa·s)					
	Valid N	Max.	Min.	Mean	Std. Dev.	Coef. Var. (%)
**60 °C**						
0.0	9	375.9	366.8	372.9	3.42	0.92
0.2	9	359.8	338.5	347.9	6.72	1.93
0.4	9	321.1	303.3	309.0	6.63	2.14
0.6	9	288.9	259.0	275.8	8.10	2.93
**90 °C**						
0.0	9	14.5	13.6	14.0	0.36	2.57
0.2	9	14.0	13.4	13.7	0.18	1.33
0.4	9	13.8	12.3	13.1	0.44	3.42
0.6	9	13.4	12.1	12.7	0.40	3.15
**135 °C**						
0.0	9	0.667	0.611	0.649	0.02	3.05
0.2	9	0.603	0.579	0.588	0.01	1.65
0.4	9	0.531	0.509	0.521	0.01	1.86
0.6	9	0.509	0.479	0.494	0.01	2.40

**Table 7:** Evaluation of the significant influence of the SAA factor on η_60_, η_90_, η_135_ (ANOVA).

Variable	SS	MS	F	p-Value
η_60_	49321	16440	394.50	<0.001
η_90_	9.782	3.261	24.86	<0.001
η_135_	0.18337	0.06112	239.47	<0.001

**Table 8:** Statistical significance of the effects of SAA and test temperatures on dynamic viscosity of 50/70 bitumen (ANOVA).

Effect	Variable: *η* (Pa·s); R^2^ = 0.99492; R^2^ − adj = 0.99467; Pure Error MS = 13.93529			
	SS	MS	F	p-Value
(1)SAA (%)(L)	13838	13838	993.0	<0.001
SAA (%)(Q)	51	51	3.6	0.059
(2)Temp (°C)(L)	1910862	1910862	137124.0	<0.001
Temp (°C)(Q)	790462	790462	56723.8	<0.001
1L* 2L	21067	21067	1511.8	<0.001
Lack of fit	11366	1894	135.9	<0.001
Pure error	1338	14	-	-
Total SS	2502298	-	-	-

**Table 9:** Parameters of the model for the effect of SAA content and test temperature on dynamic viscosity of 50/70 bitumen.

Effect	Statistical Parameters					
	Regression Coeff.	SE	t	p-Value	−95,% Cof. Lmt	+95,% Cnf. Lmt
Intercept	1756.998	5.364843	327.502	<0.001	1746.349	1767.647
SAA (%)	−238.110	7.491880	−31.782	<0.001	−252.981	−223.238
SAA^2^ (%)	−17.105	8.980203	−1.905	0.060	−34.931	0.720
Temp (°C)	−31.341	0.113826	−275.337	<0.001	−31.566	−31.115
Temp^2^ (°C)	0.135	0.000568	238.168	<0.001	0.134	0.136
SAA (%) * Temp (°C)	2.027	0.052119	38.882	<0.001	1.923	2.130

**Table 10:** Evaluation of the effect of SAA and FWC on ER, HL, and FI of the foamed bitumen.

**Effect**	**Variable: ER (-); R^2^ = 0.9875; Adj.: 0.98406; Residual MS = 0.8758664**				
	**SS**	**df**	**MS**	**F**	**p-Value**
(1)FWC (%)(L)	736.129	1	736.1286	840.4576	<0.001
FWC (%)(Q)	7.741	1	7.7411	8.8382	0.008
(2)SAA (%)(L)	472.033	1	472.0333	538.9330	<0.001
SAA(%)(Q)	6.000	1	6.0000	6.8504	0.017
1L * 2L	26.331	1	26.3314	30.0633	<0.001
Error	15.766	18	0.8759	-	-
Total SS	1264.000	23	-	-	-
**Effect**	**Variable: HL (s); R^2^= 0.9624; Adj.: 0.95197; Residual MS = 1.61479**				
	**SS**	**df**	**MS**	**F**	**p-value**
(1)SAA (%)(L)	280.6021	1	280.6021	173.7700	<0.001
SAA (%)(Q)	0.0938	1	0.0938	0.0581	0.812
(2)FWC (%)(L)	428.7937	1	428.7937	265.5415	<0.001
FWC (%)(Q)	10.8936	1	10.8936	6.7461	0.018
1L * 2L	23.7902	1	23.7902	14.7327	0.001
Error	29.0662	18	1.6148	-	-
Total SS	773.2396	23	-	-	-
**Effect**	**Variable: FI (s); R^2^ = 0.96337; Adj: 0.95319; Residual MS = 1282.081**				
	**SS**	**df**	**MS**	**F**	**p-Value**
(1)SAA(%)(L)	474720.1	1	474720.1	370.2732	<0.001
SAA(%)(Q)	6684.3	1	6684.3	5.2137	0.035
(2)FWC (%)(L)	122235.3	1	122235.3	95.3414	<0.001
FWC (%)(Q)	596.7	1	596.7	0.4654	0.504
1L * 2L	2655.3	1	2655.3	2.0711	0.168
Error	23077.5	18	1282.1	-	-
Total SS	629969.2	23	-	-	-

**Table 11:** Parameters of the model of the relationship between ER, HL, FI, and SAA quantity and FWC.

Response	Effect	Parameter	SE	t(18)	p-Value	−95,% Cnf. Lmt	+95,% Cnf. Lmt
**ER** R^2^ = 0.9873	Intercept	2.963	2.362	1.254	0.225	−1.999	7.926
	(1)SAA [%](L)	−2.752	4.063	−0.677	0.506	−11.289	5.784
	SAA [%](Q)	12.500	4.776	2.617	0.017	2.466	22.534
	(2)FWC [%](L)	−0.169	1.726	−0.099	0.923	−3.795	3.457
	FAC [%](Q)	0.911	0.307	2.973	0.008	0.267	1.554
	1L * 2L	5.486	1.001	5.483	0.001	3.384	7.588
**HL** R^2^ = 0.9742	Intercept	26.148	3.208	8.151	<0.001	19.408	32.886
	(1)SAA [%](L)	28.693	5.518	5.201	<0.001	17.102	40.284
	SAA [%](Q)	1.563	6.484	0.241	0.812	−12.061	15.186
	(2)FWC [%](L)	−9.328	2.344	−3.980	<0.001	−14.251	−4.404
	FWC [%](Q)	1.080	0.416	2.597	0.018	0.207	1.954
	1L * 2L	−5.214	1.358	−3.838	<0.001	−8.068	−2.360
**FI** R^2^ = 0.9637	Intercept	−137.999	90.380	−1.527	0.144	−327.881	51.882
	(1)SAA [%](L)	227.147	155.463	1.461	0.161	−99.469	553.762
	SAA [%](Q)	417.219	182.723	2.283	0.034	33.333	801.104
	(2)FWC [%](L)	111.026	66.033	1.681	0.109	−27.706	249.758
	FWC [%](Q)	−7.996	11.720	−0.682	0.503	−32.619	16.628
	1L * 2L	55.087	38.278	1.439	0.167	−25.333	135.507

**Table 12:** Foamed 50/70 bitumen parameters versus binder type and additive content.

Binder type	Foaming Parameters		
	ER (-)	HL (s)	FWC (%)
50/70	10	8	2.5
50/70 + 0.2% SAA	12	10	2.5
50/70 + 0.4% SAA	17	14	2.5
50/70 + 0.6% SAA	19	21	2.5

**Table 13:** Parameters of the model of the binder properties versus SAA.

Variable	SS for The Full Model with Respect to The SS for Residuals										
	R	R^2^	Adj.-R^2^	SS Model	DF Model	MS Model	SS Resid.	Df Resid.	MS Resid.	F	p-Value
***T_R&B_***	0.3688	0.1360	0.0836	2.40	2	1.20	15.250	33	0.462	2.598	0.090
***T_Fraass_***	0.9018	0.8134	0.8020	18.88	2	9.44	4.333	33	0.131	71.927	<0.001
***η_90_***	0.8310	0.6905	0.6718	9.65	2	4.82	4.325	33	0.131	36.824	<0.001
***η_135_***	0.9750	0.9507	0.9477	0.1299	2	0.06	0.006	33	0.000	318.55	<0.001
**PI**	0.2863	0.0819	0.0263	8.73	2	4.36	97.850	33	2.965	1.473	0.243
**ER**	0.9654	0.9321	0.9280	520.64	2	260.32	37.911	33	1.148	226.59	<0.001
**HL**	0.9903	0.9807	0.9795	883.41	2	441.70	17.338	33	0.525	840.67	<0.001
